# Deep Learning–Based Automated Diagnostic Charting on Panoramic Radiography: Comparison of YOLOv11 and YOLOv12

**DOI:** 10.1007/s10266-026-01333-3

**Published:** 2026-02-16

**Authors:** Onur Mutlu, Elif Aslan, Ali Mert

**Affiliations:** 1 Faculty of Science, Department of Computer Science, Karadeniz Technical University, Trabzon, Türkiye; 2 Faculty of Dentistry, Department of Oral and Maxillofacial Radiology, Izmir Tinaztepe University, Izmir, Türkiye; 3 Faculty of Engineering and Architecture, Department of Engineering Sciences, Izmir Katip Celebi University, Izmir, Türkiye

**Keywords:** Artificial intelligence, Deep learning, Dentistry, Panoramic radiography, Computer-aided diagnosis, Clinical decision support system

## Abstract

Automated diagnostic charting on panoramic radiographs is essential for optimizing clinical workflows and reducing diagnostic variability. This study aims to perform a comparative performance analysis of two next-generation deep learning architectures, YOLOv11 and YOLOv12, for the automated detection of 13 different dental conditions. A hybrid dataset consisting of 2,297 panoramic radiographs was compiled, comprising 1,579 images from a single institution and 718 external images from Roboflow Universe to test generalization. The models were trained to detect conditions including caries, implants, bone loss, and impactions using a standardized training protocol. Performance was evaluated using mean Average Precision (mAP@0.5), Precision, Recall, and F1-score on both internal and external test sets. YOLOv11 demonstrated superior performance compared to YOLOv12, achieving a mAP@0.5 of 0.857 on the internal test set. Furthermore, YOLOv11 exhibited robust generalization capabilities, maintaining a mAP@0.5 of 0.806 on the unseen external dataset. While detection rates were high for well-defined objects like crowns and implants, performance was lower for subtle pathologies such as bone loss and caries. The findings identify YOLOv11 as the more robust and reliable architecture for multi-class dental object detection compared to the newer YOLOv12. The proposed model holds significant potential as a clinical decision support tool, serving as a reliable "second opinion" to enhance diagnostic accuracy and efficiency in daily practice.

## Introduction

Dental radiography constitutes a cornerstone of modern dentistry, serving as an indispensable diagnostic tool for the diagnosis, treatment planning, and monitoring of dental diseases [1–3]. Despite the wide-spread use of two-dimensional dental radiographs, they still pose significant challenges due to their technical limitations, including the superimposition of anatomical structures, image distortions, and artifacts [4–6]. To overcome these challenges and optimize diagnostic processes, artificial intelligence and its subfield, deep learning, have emerged as a transformative technology in the field of dental radiology [1, 7, 8]. Deep learning-based systems, particularly Convolutional Neural Networks (CNNs), offer the potential to increase diagnostic accuracy, reduce inter-clinician interpretive variability, and enhance the efficiency of clinical workflows by rapidly and consistently analyzing large volumes of radiographic data [9, 10].

The artificial intelligence based algorithms have a broad range of applications in oral and maxillofacial radiology, including the automated detection, segmentation, and numbering of teeth; the identification of dental caries [15, 16], periodontal bone loss [17, 18], periapical pathologies [10, 19], impacted teeth [20], dental restorations [7], and the classification of brands and types of dental implant systems [21–23]. In this respect, ‘You Only Look Once' (YOLO) family of architectures has become one of the foundational and well-established technologies widely used in the dentistry, particularly for object detection, due to its ability to combine speed and accuracy [9, 14, 20]. Previous studies employing different architectures of the YOLO family for the detection of various dental conditions on panoramic images have reported notably high performance rates indicating the reliability of these architectures as a supporting tool in daily clinical practice [24–28]. Akgül et al. demonstrated sensitivity, precision, and F1-score values exceeding 0.90 for detecting fillings on panoramic images, while Ayhan et al. reported performance metrics above 0.80 for identifying dental caries under fixed dental prostheses. Accordingly, the YOLO family has been proven to be a highly adaptable and effective architecture for dental image analysis, capable of accurately detecting diverse pathologies and restorative conditions across different datasets.

Panoramic radiography is one of the most indispensable imaging modalities in dentistry due to its ability to capture the entire dentoalveolar complex in a single exposure and its routine integration into clinical workflows for initial assessment. As a widely employed radiographic technique, panoramic radiography facilitates diagnostic charting and baseline screening by providing a fast, comprehensive overview of teeth, restorations, and prevalent pathologies [29]. However, despite this potential of panoramic images, most previous studies have focused only on a limited subset of dental conditions [24, 25, 27, 28], while the development of a comprehensive diagnostic charting based on panoramic radiographic images has rarely been addressed [30–32]. Moreover, even though different AI-based architectures have been proven to be effective for the detection of many dental pathologies and conditions, there remains no study to guide clinicians on which state-of-the-art model currently delivers the highest performance for diagnostic charting on panoramic images.

In this context, the primary aim of this study is to address this gap by comparatively evaluating the performance of two next-generation deep learning architectures, YOLOv11 [33] and YOLOv12 [34], for the automated detection of 13 different dental conditions on digital panoramic images. The present study seeks to identify the more robust and accurate architecture to serve as a foundation for a comprehensive and reliable support tool for automated diagnostic charting.

## Materials and methods

### Ethical approval

This study was approved by the Medical Research Ethics Committee (Date: 10.07.2025, No: 25-7 T/23) of the university and followed the principles of the Declaration of Helsinki.

### Methodological framework

This study adopted a systematic methodological framework structured into three primary phases. The workflow commenced with dataset acquisition and curation, followed by a rigorous data management process that included data polishing, denoising, and enhancement to ensure optimal input quality. Subsequently, the preprocessed dataset was utilized to train and comparatively evaluate the YOLOv11 and YOLOv12 deep learning architectures for the automated detection of dental conditions. The final stage involved a comprehensive performance analysis using a diverse set of metrics to assess both the internal accuracy and the generalization capabilities of the models. Each of these stages is detailed in the following sections.

### Dataset acquisition and annotation protocol

The dataset for this study was compiled from two distinct sources. The primary source consisted of digital panoramic radiography (DPR) images archived by the Department of Oral and Maxillofacial Radiology between 2023 and 2024. From the initial random selection of 4,000 DPRs, images meeting exclusion criteria—including primary or mixed dentition, edentulous cases, persistent deciduous teeth, intra-osseous pathologies, or severe artifacts—were removed, resulting in a final dataset of 1,579 clinically acquired images. To augment and diversify the dataset, additional DPRs were obtained from a publicly available collection hosted on Roboflow Universe. Following the application of necessary preprocessing steps and a manual verification of both images and annotations to ensure suitability, a total of 718 externally acquired images were retained for the study. Consequently, a total of 2297 DPRs from patients with permanent dentition were anonymized and used in the study.

One thousand five hundred seventy-nine internally collected DPRs had been obtained using a single Kodak 8000 device (Carestream Health Inc., Trophy, France) with standardized exposure parameters (68 kV, 8 mA, 13.9 s) and an original image size of 2943 × 1435 pixels. The internal images were manually labeled by an oral and maxillofacial radiologist (EA, five years of clinical experience) using Colabeler annotation software (MacGenius, Blaze Software, CA). Annotations for the 718 external images were adopted following verification by the same radiologist.

Diagnostic charting included thirteen different dental conditions: Attrition, bone loss, braces, caries, crown, filling, fractured tooth, furcation, impacted tooth, implant, root canal treated, root pieces and unerupted tooth. The radiologist was instructed to draw bounding boxes around the crown, root or entire tooth and to assign an appropriate class label to each box. Care was taken to draw a bounding box of minimum size for each dental condition in all PR images. While only the crown of the tooth was included in the bounding box for the labels of attrition, braces, caries, crown and filling, the bounding box covered only the root for root canal treated and root pieces. For impacted and unerupted teeth, the entire tooth was selected. Furcation regions of multirooted teeth exhibiting radiographically visible bone loss were labeled as furcation. Interdental bone areas showing horizontal or vertical bone loss, as determined with reference to the cemento-enamel junction, were labeled as bone loss. Table 1 presents the number of annotations made for each dental condition.

**Table 1:** The number of annotations made for each dental condition in the internal and external datasets

Dental condition	Internal dataset	External dataset	Total
Attrition	764	155	919
Bone loss	3,959	1,612	5,571
Braces	135	63	198
Caries	2,111	677	2,788
Crowns	2,397	1,660	4,057
Filling	5,110	1,799	6,909
Fractured tooth	24	30	54
Furcation	126	62	188
Impacted	1,661	498	2,159
Implant	108	381	489
Root-canal treated	1,047	959	2,006
Root pieces	232	412	644
Unerupted	460	563	1,023

### Data preparation, splitting, and augmentation

A strategic data partitioning protocol was implemented to ensure a robust evaluation of the models' generalization capabilities across different data sources. The internally collected dataset of 1579 images was exclusively used for model development and validation. This dataset was partitioned into a training set (85%, 1342 images), a validation set (10%, 158 images), and an initial test set (5%, 79 images). To rigorously assess the models' performance on unseen data from a different domain, the final test set was constructed as a hybrid collection. It combined the initial test split of 79 internal images with the entire external dataset of 718 images from Roboflow, creating a comprehensive and challenging final test set of 797 images. Using this approach, it was aimed to ensure that the models are trained and validated on a consistent, single-source dataset while being tested against both familiar and entirely novel data distributions.

To enhance the models' generalization capabilities and prevent overfitting, various data augmentation techniques were applied to the training set. These strategies were selected to digitally simulate common image variations encountered in clinical practice. The primary strategies implemented were as follows:

- Geometric Augmentation: Random rotation between −5 and + 5 degrees, random scaling between 90 and 110%, and horizontal flipping with a 50% probability.
- Photometric Augmentation: Random changes to the brightness, contrast, and saturation values of the images within a ± 20% range.
- Noise Addition: Gaussian noise was randomly added to the images in the training set to make the models more resilient to minor imperfections and sensor noise.

### Deep learning framework for automated dental condition detection

In the present study, the performance of two contemporary architectures, YOLOv11 and YOLOv12, which represent different optimization philosophies in the field, was comparatively analyzed. YOLO is a single-stage detector architecture considered a revolution in the field of object detection. Unlike two-stage approaches such as the R-CNN (Regions with Convolutional Neural Networks) family, YOLO processes an image in a single neural network pass. Accordingly, instead of first identifying potential object regions and then classifying them, this approach formulates bounding box regression and class prediction as a single, unified task, resulting in high-speed performance making YOLO ideal for real-time applications.

The YOLOv11 architecture is built upon the proven modular structure of previous versions, with a design that focuses particularly on flexibility and scalability. Its architecture consists of three main components: Backbone, Neck, and Head. The Backbone, a deep network based on Cross Stage Partial (CSP) design, extracts rich semantic features from the image. In the Neck layer, a Path Aggregation Network (PANet) structure is used to combine these features from different resolution levels and to enrich contextual information. This structure enhances the model's capacity to detect both small and large pathologies simultaneously with high accuracy. Finally, the Head section generates the final detections from these fused feature maps.

YOLOv12, in contrast, adopts a different design philosophy, focusing specifically on inference efficiency and hardware acceleration. Its architecture includes innovations such as a lighter yet powerful backbone, advanced feature fusion networks, and a modified loss function aimed at improving the detection of small objects. These design choices make YOLOv12 a potentially faster and less computationally demanding alternative, while aiming to maintain accuracy without compromise.

### Experimental setup and training strategy

#### Experimental setup

All deep learning experiments were conducted on a high-performance computing workstation. The hardware configuration consisted of an NVIDIA GeForce RTX 3090 GPU (24 GB GDDR6X VRAM), an AMD Ryzen 9 5900X CPU, and 64 GB of DDR4 3600MHz system memory. The software environment comprised the Ubuntu 22.04 operating system, Python 3.11, the PyTorch v2.5.1 deep learning library, and the CUDA v12.6 parallel computing platform.

#### Training strategy

To establish a rigorous and unbiased comparison between the YOLOv11 and YOLOv12 architectures, the 'Extra Large (x)' variant was selected for both models, ensuring equivalence in computational complexity and parameter count. Furthermore, a unified training protocol was strictly implemented for both architectures. The comprehensive set of hyperparameters, selected through preliminary experimentation, is detailed in Table 2. Optimization was performed using Stochastic Gradient Descent (SGD). The optimizer was enhanced with momentum and weight decay to accelerate convergence and mitigate overfitting, respectively. A dynamic learning rate schedule was employed, initiating with a warm-up phase to prevent early gradient divergence, followed by a cosine annealing scheduler. The entire learning process was guided by a composite loss function that optimized for bounding box regression accuracy, object confidence, and classification correctness.

**Table 2:** Hyperparameter configuration for model training

Parameter	Value	Description
Input size	1280 × 1280	The resolution to which all images are resized, balancing computational load and feature preservation
Batch size	4	The number of images processed simultaneously in each training step, selected to optimize GPU memory utilization
Number of epoch	150	The total number of times the entire training dataset is passed through the model to ensure full convergence
Optimizer	SGD	The Stochastic Gradient Descent (SGD) algorithm used to update the model’s weights during the learning process
Learning rate	0.01	The initial coefficient that determines the step size for updating the model's weights
IoU threshold	0.5	The minimum Intersection over Union (IoU) ratio required for a prediction to be considered a positive match during training

### Performance evaluation metrics

The performance of the trained models was quantitatively assessed using a comprehensive set of metrics. The loss functions used to guide model optimization and the metrics used to evaluate final performance are detailed in Table 3.

**Table 3:** Loss Functions and Performance Evaluation Metrics

Category	Metric	Formula	Description
Loss functions	Box Loss (${L}_{box}$):	$\frac{1}{N} \sum_{i=1}^{N}\Vert {\widehat{b}}_{i}- {b}_{i}\Vert$	Measures the geometric discrepancy between the predicted bounding box and the ground-truth box
	Class Loss (${L}_{cls}$):	$-\frac{1}{N} \sum_{i=1}^{N}\sum_{c=1}^{C}{y}_{i}\mathrm{log}({\widehat{y}}_{i,c})$	A cross-entropy loss that measures the model's ability to assign the correct class label to a detected object
	Distribution Focal Loss (${L}_{DFL}$):	$-\frac{1}{N} \sum_{i=1}^{N}\alpha {\left(1-{p}_{i}\right)}^{\gamma }\mathrm{log}{(p}_{i})$	A specialized focal loss that optimizes the probability distribution of bounding box locations, improving precision for ambiguous objects
Fundamental metrics	Accuracy	$\frac{TP+TN}{TP+TN+FP+FN}$	The ratio of all correct predictions (both positive and negative) to the total number of samples
	Precision	$\frac{TP}{TP+FP}$	The ratio of correct positive detections to all predicted positive detections. High precision indicates a low false positive rate
	Recall	$\frac{TP}{TP+FN}$	The ratio of correct positive detections to all actual positive cases (ground-truth). High recall indicates a low false negative (miss) rate
	F1-Score	$2* \frac{Recall* Precision}{Recall+Precision}$	The harmonic mean of Precision and Recall, providing a single, balanced score. It is particularly useful for uneven class distributions
Object detection metrics	Average Precision (AP)	AP = ${\int }_{0}^{1}p\left(r\right)dr$	The area under the Precision-Recall curve for a single class, summarizing its performance across all recall levels
	mean Average Precision (mAP)	mAP = $\frac{1}{N}$ $\sum_{i=1}^{N}A{P}_{i}$	The gold standard for object detection, representing the mean of the AP values across all N classes to holistically summarize classification and localization performance. The mean Average Precision computed at an IoU threshold of 0.5 is defined as ‘mAP@0.5

All evaluation metrics are derived from the four components of the confusion matrix: True Positive (TP) (a correct detection of a positive class), False Positive (FP) (an incorrect detection, i.e., a predicted condition that is not present), True Negative (TN) (a correct identification of an absence of a condition), and False Negative (FN) (a missed detection, i.e., an actual condition that was not detected).

### Intra-examiner agreement

Manually labeling of panoramic images was performed by a single oral and maxillofacial radiologist (EA) with five years of clinical experience. For intra-examiner agreement, 200 panoramic radiographs from the internal dataset were randomly selected and re-labelled after a 1-month interval [28]. The intra-examiner reliability was evaluated using Cohen’s kappa statistics.

## Results

### Learning dynamics and validation set performance analysis

**Fig. 1:**
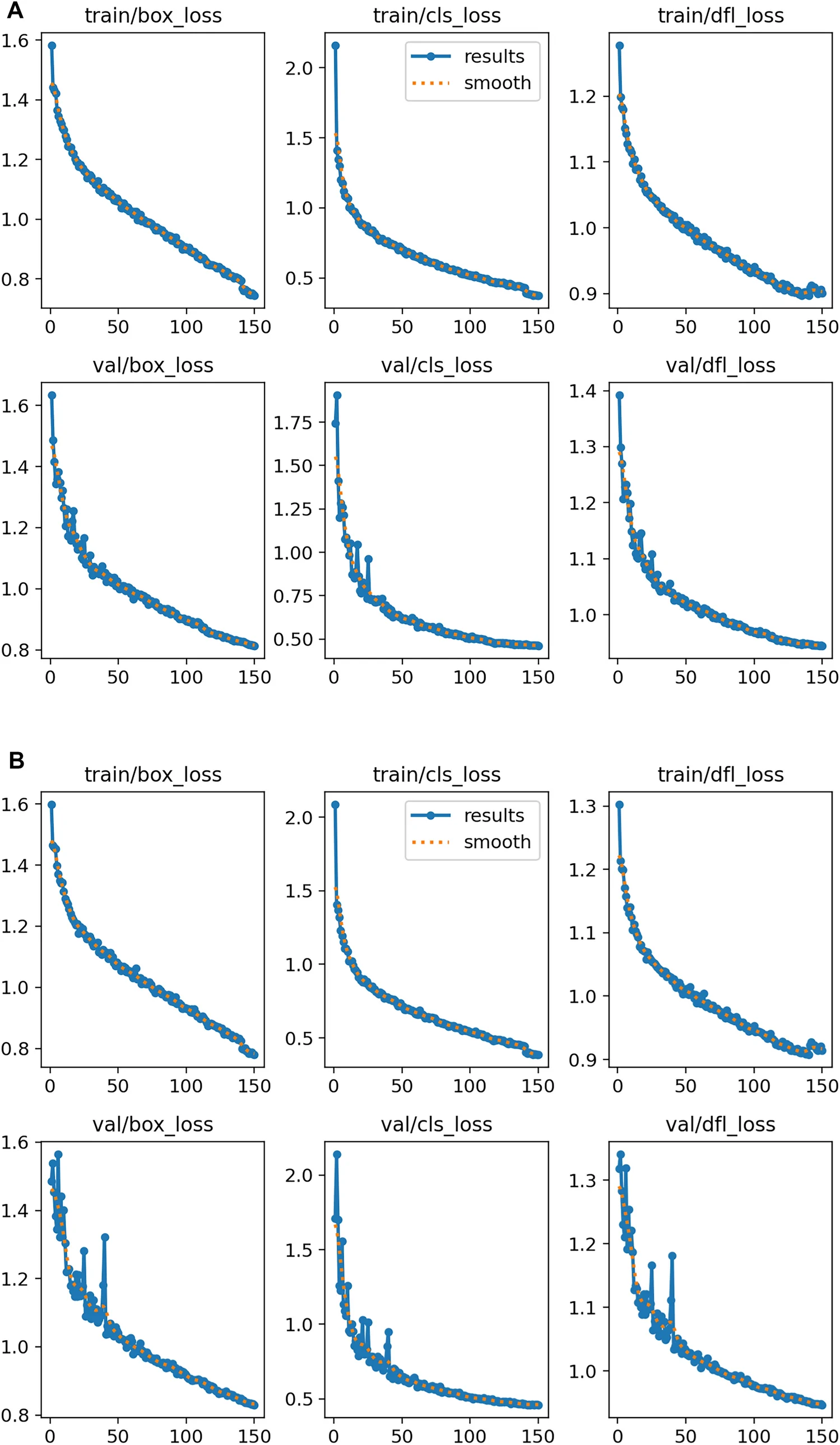
Change in the loss function of the YOLOv11 and YOLOv12 models on the training and validation sets

The learning dynamics of the models were monitored over 150 epochs by tracking three key metrics on both the training and validation sets: ${L}_{box}$, ${L}_{cls}$, and ${L}_{DFL}$. As illustrated in Fig. 1, all loss components for both architectures exhibited a consistent downward trend as training progressed. The loss values decreased sharply during the initial epochs and gradually stabilized in the later stages, indicating a stable learning process without signs of divergence between the training and validation sets.

The quantitative performance of the YOLOv11 and YOLOv12 models on the validation set is presented in Table 4. For the YOLOv11 architecture, the overall precision, recall, F1-score, and mAP@0.5 were recorded as 0.868, 0.831, 0.849, and 0.863, respectively. The corresponding aggregate values for the YOLOv12 architecture were 0.812, 0.809, 0.81, and 0.824. At a class-specific level, the AP@0.5 for YOLOv11 ranged from a maximum of 0.99 for the 'Crowns' class to a minimum of 0.516 for the 'Fractured Tooth' class.

**Table 4:** Comparative detection performance of YOLOv11 and YOLOv12 models on the verification set by class

Dental condition	YOLOv11				YOLOv12			
	Precision	Recall	F1-score	AP@.5	Precision	Recall	F1-score	AP@.5
Attrition	0.813	0.892	0.851	0.909	0.744	0.853	0.795	0.894
Bone loss	0.786	0.570	0.661	0.620	0.772	0.532	0.630	0.596
Braces	0.641	1.000	0.781	0.968	0.602	1.000	0.752	0.884
Caries	0.881	0.697	0.778	0.813	0.709	0.728	0.718	0.800
Crowns	0.952	0.963	0.957	0.990	0.912	0.895	0.903	0.989
Filling	0.943	0.937	0.940	0.960	0.934	0.924	0.929	0.961
Fractured tooth	0.743	0.541	0.626	0.516	0.608	0.473	0.532	0.242
Furcation	0.774	0.594	0.672	0.641	0.654	0.520	0.579	0.583
Impacted	0.952	0.974	0.963	0.967	0.949	0.954	0.951	0.960
Implant	0.975	0.950	0.962	0.989	0.972	0.950	0.961	0.989
Root canal treated	0.952	0.867	0.908	0.963	0.940	0.900	0.920	0.957
Root pieces	0.932	0.862	0.896	0.908	0.897	0.840	0.868	0.884
Unerupted	0.944	0.953	0.948	0.979	0.866	0.950	0.906	0.972
Average	0.868	0.831	0.849	0.863	0.812	0.809	0.81	0.824

**Fig. 2:**
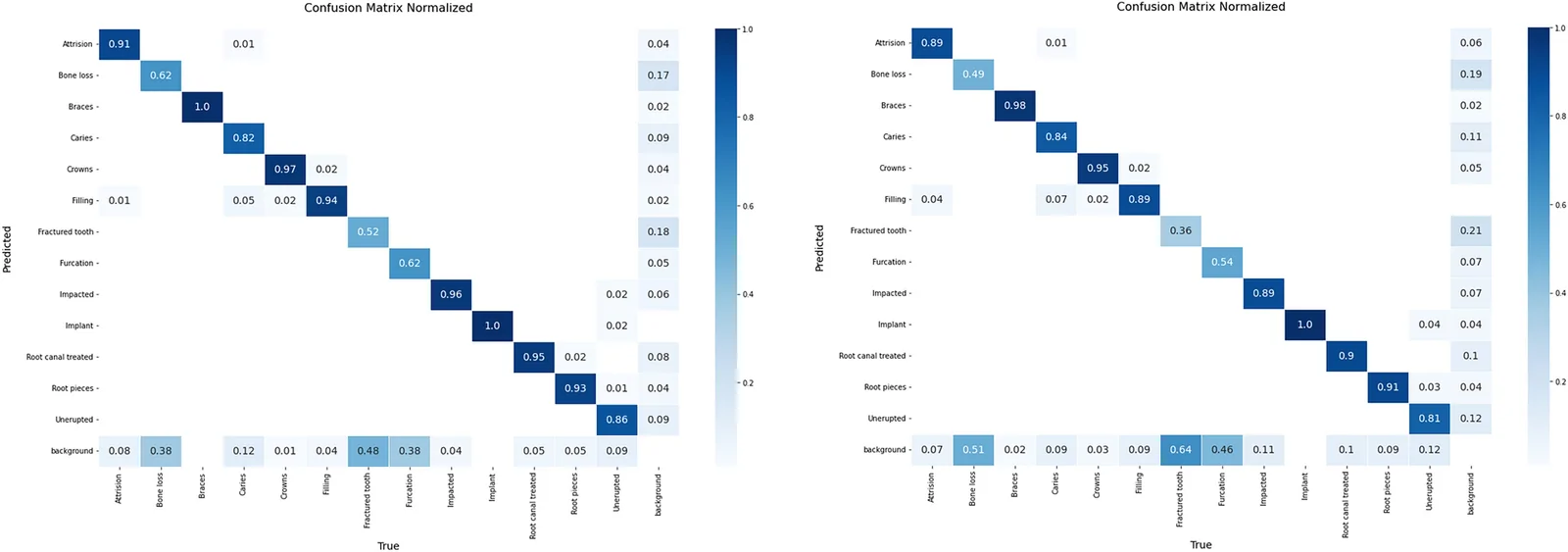
Comparative visual evaluation of detection outputs of YOLOv11 and YOLOv12 architectures

The normalized confusion matrices for both models are provided in Fig. 2, illustrating the per-class true positive rates (recall) on the diagonal. The YOLOv11 matrix demonstrated the highest recall of 1.0 for 'Braces' and 'Implant', and the lowest recall of 0.52 for 'Fractured Tooth'. For this latter class, the remaining 48% of instances were not detected by the model, constituting the false negatives. In comparison, the matrix for YOLOv12 revealed a recall of 0.36 for 'Fractured Tooth', with the remaining 64% of instances being missed by the detector. For the majority of classes, the values on the diagonal of the YOLOv11 matrix are quantitatively higher than those recorded in the YOLOv12 matrix.

### Final performance and test set results

The generalization capability of the final YOLOv11 model was evaluated on a hybrid test set composed of both internal (institution-specific) and external (publicly sourced) data. The detailed class-specific performance metrics for both components of the test set are presented in Table 5.

**Table 5:** Generalization performance of the YOLOv11 model on the test datasets

Dental condition	Internal test set				External test set			
	Precision	Recall	F1-Score	AP@.5	Precision	Recall	F1-Score	AP@.5
Attrition	0.763	0.872	0.814	0.899	0.993	0.692	0.816	0.829
Bone loss	0.696	0.71	0.703	0.61	0.646	0.52	0.576	0.653
Braces	0.633	0.94	0.757	0.818	0.841	0.984	0.907	0.898
Caries	0.854	0.837	0.845	0.893	0.641	0.627	0.634	0.753
Crowns	0.792	0.983	0.877	0.98	0.952	0.913	0.932	0.95
Filling	0.923	0.957	0.94	0.83	0.763	0.847	0.803	0.756
Fractured tooth	0.743	0.721	0.732	0.595	0.633	0.601	0.617	0.706
Furcation	0.664	0.654	0.659	0.811	0.774	0.594	0.672	0.631
Impacted	0.975	0.944	0.959	0.907	0.772	0.954	0.853	0.887
Implant	0.865	0.897	0.881	0.909	0.675	0.73	0.701	0.789
Root-canal treated	0.972	0.937	0.954	0.953	0.902	0.787	0.841	0.763
Root pieces	0.852	0.902	0.876	0.988	0.772	0.842	0.805	0.918
Unerupted	0.974	0.923	0.948	0.949	0.854	0.883	0.868	0.939
Average	0.824	0.867	0.842	0.857	0.786	0.767	0.771	0.806

Overall, the model demonstrated robust performance on the internal test set, achieving an average precision of 0.824, recall of 0.867, F1-score of 0.842, and mAP@0.5 of 0.857. The performance on the external test set was slightly lower, with an average precision of 0.786, recall of 0.767, F1-score of 0.771, and mAP@0.5 of 0.806.

A class-specific analysis of dental conditions in the internal dataset revealed that the model achieved the highest performance in detecting “Impacted” (F1-score: 0.959), “Root-canal treated” (F1-score: 0.954), and “Unerupted” (F1-score: 0.948) teeth, whereas the lowest metrics were observed for “Furcation” (F1-score: 0.659), “Bone loss” (F1-score: 0.703), and “Fractured tooth” (F1-score: 0.732). On the external dataset, the model exhibited strong performance for “Crowns” (F1-score: 0.932) and “Braces” (F1-score: 0.907), whereas “Bone loss” (F1-score: 0.576) and “Caries” (F1-score: 0.634) demonstrated the lowest metrics.

**Fig. 3:**
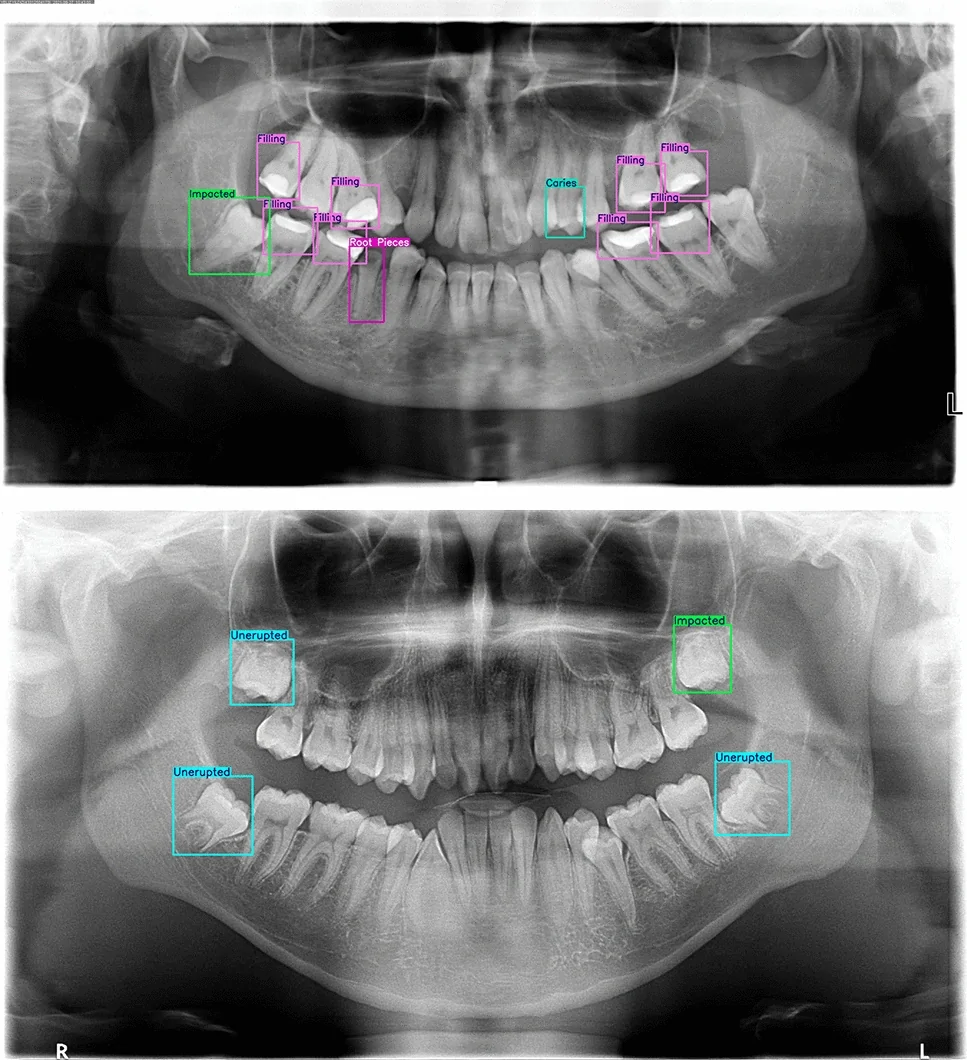
Representative performance examples of the YOLOv11 model on the test set. **A** An example of multiple true positive detections on a single panoramic radiograph, where the model correctly identified instances of 'Impacted', 'Caries', 'Root Pieces', and 'Filling'. **B** An example of a false positive due to a classification error, where the model has incorrectly labeled an 'Unerupted' tooth as 'Impacted'

Figure 3 displays representative visual examples of the YOLOv11 model's detection outputs on the test set. The figure is selected to illustrate both a successful detection case with multiple true positives in a single image (Fig. 3A), and an example of a typical failure mode a false positive resulting from a classification error (Fig. 3B).

### Intra-examiner agreement

To assess the intra-examiner reliability, analysis findings were evaluated based on 1,694 re-labeled dental condition derived from 200 randomly selected panoramic images within the internal dataset. Cohen’s kappa analysis demonstrated a statistically highly significant and “almost perfect” intra-examiner agreement (κ = 0.976, *N* = 1,694, *p* < 0.001).

## Discussion

Numerous studies have demonstrated the applicability of deep learning models for the detection of various dental conditions. However, while most existing studies have focused on isolated dental conditions or limited clinical scenarios, a substantial gap persists regarding how state-of-the-art architectures perform when applied to a comprehensive, multi-condition diagnostic charting framework. Moreover, despite continuous architectural advancements, there has been a lack of direct comparative evaluations of the newest versions to guide clinicians and researchers in selecting the most reliable model for AI-supported detection of dental conditions on panoramic images. Therefore, this study aimed to provide a rigorous comparison of two next-generation object detection architectures, YOLOv11 and YOLOv12, across 13 different dental conditions on panoramic images, in order to identify the superior model and determine its overall detection performance.

The primary findings of the present study highlighted the superior performance of the YOLOv11 architecture over YOLOv12, particularly for the detection of subtle and challenging lesions, thereby establishing YOLOv11 as the more robust and reliable model for this comprehensive detection task. The final YOLOv11 model achieved an overall mAP@0.5 score of 85.7% on the internal test set, which was higher than the performance reported in a previous study [11]. However, a direct comparison with earlier diagnostic charting research was not feasible, as most prior studies did not report an overall performance metric. [30–32, 35]. Nevertheless, the present results are consistent with similar multi-task detection studies, supporting the potential of AI-based systems for addressing complex diagnostic challenges in dental radiology [11, 23, 36], and further indicating that the YOLOv11 model, when appropriately trained, holds considerable promise as a supportive tool in routine clinical practice. Moreover, the final YOLOv11 model demonstrated an overall mAP@0.5 score of 0.806 on previously unseen external test data. The 0.06 decrease in our model's performance on the test set compared with the validation set (mAP@0.5: 0.863) is both expected and acceptable, indicating that the model did not overfit during training and is capable of generalizing effectively to previously unseen data. This score is substantially higher than that reported in a similar study by Muresanu et al. [11], who achieved a mAP@0.5 of 0.451 on an external validation set using YOLOv8 model. This significant performance improvement is likely attributable to the architectural advancements of the YOLOv11 model over earlier versions, as well as the use of a meticulously curated and high-quality training dataset. This result also shows that the more recent YOLO versions offer improved generalizability in real-world clinical datasets, suggesting that these architectures may be used more reliably in clinical planning. However, this inference requires further validation through studies conducted on larger and more diverse external datasets. Nevertheless, given that this study utilizes one of the newest architectures alongside a sufficiently large dataset, the reported performance likely reflects the current upper boundary of what state-of-the-art AI-based diagnostic support systems can achieve on DPRs. Despite these advancements, the present results also indicate that automated detection models still fall short of capturing the full diversity and complexity of dental diseases at a level comparable to specialists, underscoring the ongoing need for methodological improvements and more comprehensive datasets.

Consistent with previous reports, the class-based analysis of dental conditions revealed a notable performance disparity among targets characterized by subtle or morphologically variable features. Both YOLO models demonstrated higher efficacy in detecting targets with well-defined boundaries and pronounced radiographic contrast, such as “Crowns,” “Implants,” and “Fillings” [5, 9, 21]. This finding is likely attributable to the consistent and distinct morphological features of these dental conditions, which provide strong and unambiguous signals for the detection algorithm. On the other hand, dental pathologies or conditions characterized by subtle and morphologically variable features with low radiographic contrast, such as “Bone loss,” “Furcation,” “Fractured tooth,” and “Caries,” exhibited the lowest performance metrics in both models. Although the number of “Bone loss” cases in our dataset was relatively sufficient, its detection performance remained low, likely due to the wide morphological variability of bone loss, which makes capturing a consistent radiographic pattern challenging for the model. This finding was not only consistent with the results of previous studies [17, 20, 35] but also supports the notion that lesions with low contrast relative to surrounding anatomical structures, as well as the inherent ambiguity in their radiographic presentation, may be considerably more challenging for AI-supported detection models [11]. Furthermore, the low number of samples for “Fractured tooth” and “Furcation” likely contributed to their reduced detection performance, whereas the “Implant” class demonstrated consistently high detection accuracy despite a relatively smaller sample size, suggesting that strong and consistent radiographic patterns can compensate for limited sample availability. It should be also considered that panoramic images may hinder the accurate placement of bounding boxes due to inherent limitations such as superposition and distortion, which can contribute to reduced detection performance. To address this challenges, Hua et al. recommended integrating lightweight C2f-Faster blocks, BiFPN-based multi-scale feature fusion, an enhanced EMCA attention mechanism, and the Powerful-IoU loss to enhance the representation of subtle lesion characteristics, and increase the precision of localization [29]. Their improved architecture demonstrated superior identification of small, low-contrast findings such as caries and deep caries, and therefore offers considerable promise for future studies [29]. Still, our model achieved an F1-score of 0.845 for caries detection on the internal test set and an acceptable F1-score of 0.634 on the external test set. This performance demonstrates the robustness of the applied training pipeline and suggests that well-structured data preprocessing, augmentation, and class-balancing strategies can partially compensate for some of the inherent limitations of panoramic imaging. This performance demonstrates the robustness of the applied training pipeline, suggests the combination of that well-structured data preprocessing, augmentation techniques, and the architecture’s inherent loss functions designed to mitigate class imbalance can partially compensate for the limitations imposed by the 2D nature of panoramic imaging. However, despite these strengths, the overall F1-score of 0.842 on the internal dataset (0.771 on the external dataset) indicates that although the model's multi-condition diagnostic capability is promising, it clearly requires further improvements, particularly given the suboptimal detection performance in clinically critical conditions such as bone loss and caries. Accordingly, continued improvements in both model architecture and dataset diversity may enhance the model’s detection ability, ultimately enabling more reliable and comprehensive diagnostic support. In addition, while this study adopted a multi-task learning framework, future research may explore the benefit of training separate, task-specific models that focus on individual dental conditions, which may potentially improve detection performance for subtle or clinically important findings.

Our study demonstrated that the proposed YOLOv11-based model can achieve reliable diagnostic performance across multiple dental conditions. Nonetheless, certain limitations should be taken into account when interpreting the present findings. While our final test set included an external dataset to better assess generalization, the training and validation data were sourced from a single institution using a single type of digital panoramic radiography device. Although this ensures internal consistency, the lack of multi-center training data may limit the model's robustness to variations from different devices and patient populations [9, 22, 23]. A second limitation is that the ground truth for the internal dataset was established by a single radiologist. Although the reliability of dental condition labeling was demonstrated by high intra-examiner agreement, the absence of a second examiner for validation and the lack of inter-examiner agreement analysis indicate that the potential for individual bias in the annotations cannot be entirely excluded. Finally, due to the random selection of panoramic images, an equal data distribution across different dental conditions could not be achieved. Therefore, classes such as “Fractured tooth,” “Braces,” “Implants,” and “Furcation” were represented by a smaller number of samples compared to the other dental conditions, which may have influenced the overall results. In addition, the relatively lower detection performance in critical treatment-requiring conditions such as bone loss and caries should be considered a key limitation, emphasizing that current AI models may miss clinically significant findings and therefore should be used cautiously in real-world clinical workflows.

Despite these limitations, the findings of the current study offer significant implications for the potential role of AI-based systems in clinical practice. The system we developed can serve as a "second opinion" or a pre-screening tool, especially in dental hospitals with a rapid workflow and large patient load [1, 2, 36]. The detection model can also accelerate the diagnostic workflow, particularly by rapidly identifying common and distinct dental conditions [9, 12]. However, the model's lower performance on certain dental conditions and its potential for error strongly underscore that such systems should be integrated as supportive tools to assist rather than replace the clinician’s final judgment [9, 22]. With further refinement, AI-based supporting models may also offer practical utility for automated dental charting during initial patient visits by providing rapid and standardized preliminary assessments. Moreover, such models may hold significant value in educational settings by serving as structured training tools—including preclinical teaching modules, simulation-based radiographic interpretation exercises, and decision-support tools integrated into educational software platforms—to assist dental students in systematically learning to identify radiographic abnormalities.

## Conclusion

This study successfully conducted a comparative analysis of two modern object detection architectures, YOLOv11 and YOLOv12, for the automated detection of 13 dental conditions on panoramic radiographs. The findings demonstrated that the YOLOv11 architecture exhibits significantly superior performance, particularly in the identification of subtle and morphologically challenging lesions. The results underscore the potential of this high-performing model to serve as a valuable "second opinion" for clinicians, enhancing diagnostic consistency and accelerating clinical workflows. However, the performance variations across different dental conditions confirm that this technology should be positioned as a decision support tool rather than a substitute for clinical expertise. Future work should focus on validating the model's robustness and generalization capabilities using larger, multi-center datasets to further advance its potential for clinical integration.

## Data Availability

The external dataset supporting the findings of this study ("Tooth" dataset by Tomasz Derek) is openly available in Roboflow Universe at https://universe.roboflow.com/tomasz-derek/tooth-cwxhl. The internal data that support the findings of this study are available from the corresponding author upon reasonable request.
